# Polyploid/Multinucleated Giant and Slow-Cycling Cancer Cell Enrichment in Response to X-ray Irradiation of Human Glioblastoma Multiforme Cells Differing in Radioresistance and TP53/PTEN Status

**DOI:** 10.3390/ijms24021228

**Published:** 2023-01-08

**Authors:** Lina Alhaddad, Roman Chuprov-Netochin, Margarita Pustovalova, Andreyan N. Osipov, Sergey Leonov

**Affiliations:** 1 School of Biological and Medical Physics, Moscow Institute of Physics and Technology, 141700 Dolgoprudny, Russia; 2 State Research Center-Burnasyan Federal Medical Biophysical Center of Federal Medical Biological Agency (SRC-FMBC), 123098 Moscow, Russia; 3 N.N. Semenov Federal Research Center for Chemical Physics, Russian Academy of Sciences, 119991 Moscow, Russia; 4 Institute of Cell Biophysics, Russian Academy of Sciences, 142290 Pushchino, Russia

**Keywords:** glioblastoma multiforme, radioresistance, senescence, multinucleated giant cancer cell (MGCC), polyploid giant cancer cell (PGCC), p53, p63, p73, neosis

## Abstract

Radioresistance compromises the efficacy of radiotherapy for glioblastoma multiforme (GBM), the most devastating and common brain tumor. The present study investigated the relationship between radiation tolerance and formation of polyploid/multinucleated giant (PGCC/MGCC) and quiescent/senescent slow-cycling cancer cells in human U-87, LN-229, and U-251 cell lines differing in *TP53/PTEN* status and radioresistance. We found significant enrichment in MGCC populations of U-87 and LN-229 cell lines, and generation of numerous small mononuclear (called Raju cells, or RJ cells) U-87-derived cells that eventually form cell colonies, in a process termed neosis, in response to X-ray irradiation (IR) at single acute therapeutic doses of 2–6 Gy. For the first time, single-cell high-content imaging and analysis of Ki-67- and EdU-coupled fluorescence demonstrated that the IR exposure dose-dependently augments two distinct GBM cell populations. Bifurcation of Ki-67 staining suggests fast-cycling and slow-cycling populations with a normal-sized nuclear area, and with an enlarged nuclear area, including one resembling the size of PGCC/MGCCs, that likely underlie the highest radioresistance and propensity for repopulation of U-87 cells. Proliferative activity and anchorage-independent survival of GBM cell lines seem to be related to neosis, low level of apoptosis, fraction of prematurely stress-induced senescent MGCCs, and the expression of p63 and p73, members of p53 family transcription factors, but not to the mutant *p*53. Collectively, our data support the importance of the *TP53wt/PTENmut* genotype for the maintenance of cycling radioresistant U-87 cells to produce a significant amount of senescent MGCCs as an IR stress-induced adaptation response to therapeutic irradiation doses.

## 1. Introduction

Glioblastoma multiforme (GBM) is the most lethal and aggressive primary brain tumor that accounts for >60% of all brain tumors, with a median survival of approximately 12–15 months [1,2,3,4]. Since radiotherapy (RT) remains a significant treatment modality for gliomas, the radioresistance of glioma cells and targets to modify their radiation tolerance are of significant interest. The lack of access to resistant cells from patients undergoing radiotherapy is a drawback in understanding cellular and molecular events underlying radioresistance and relapse in GBM. The inability to recognize the changes in the resistant cells during the treatment hampers our understanding of the radioresistance mechanism in GBM. As a result, GBM remains incurable, and relapse is almost inevitable in GBM patients, meaning treatment of GBM is a significant challenge. GBM stem cells (GSCs) contribute to GBM chemo- and radiotherapeutic treatment resistance and enhance tumor propagation [5]. It has been thought that GSC elimination from the bulk tumor could result in inability of the tumor to sustain itself and disseminate [6].

Over the past decade, plentiful evidence indicated that tumor polyploid/multinucleated giant cancer cells (PGCCs/MGCCs) significantly contribute to tumor cell heterogeneity and fate in metastasis, therapy resistance, and disease relapse. Irradiation can noticeably augment the MGCCs’ frequency. Albeit PGCCs/MGCCs are dormant, they stay metabolically active and promote the stemness of neighboring cells [1]. MGCCs were consistently seen in human cancer tissues and cell lines primarily associated with late stages of tumors [7,8]. Few reports indicated the formation of the multinucleated cells due to radiation, although these cells experienced cell death by mitotic catastrophe [9,10]. Pre-existing MGCCs cause resistance to therapies [11,12]. Moreover, the proportion of MGCCs both in vitro and in vivo increases markedly under stressful conditions due to replicative stress [13] and hypoxia [12,14], which also occurs after exposure of cancer cells, particularly GBM, to ionizing radiation (IR) [15,16,17]. The question remains whether radioresistance is a cause or consequence of either de novo formed or pre-existing PGCCs/MGCCs, respectively.

Several pieces of evidence suggest that after chemo- and radiotherapeutic treatment, cancer cells often enter a dormant state accompanied by cell size increase and giant cell development with either a highly enlarged nucleus or multiple nuclei, reflecting stress-induced premature senescence (SIPS) [5,6,18,19]. These PGCCs/MGCCs remain viable for long times post-treatment [20,21,22] and contribute to cancer relapse, leading to progeny with stem cell-like property [23,24,25]. The fraction of these cells amounts to 0.1–20% of the total population, depending on the tumor localization and stage [24]. MGCCs may derive from cell fusion or cell division [25]. 

The source of dormant cancer cells in tumor relapse remains obscure. The dormant cancer cells escape treatment by avoiding S phase, which is often the target of chemotherapy and radiotherapy [26]. Indeed, the fraction of dormant cancer cells increases due to chemotherapy using cisplatin [27], doxorubicin [27,28,29], paclitaxel [27,30], 5-fluouracil [31], gemcitabine [32], or radiation [29]. Whether these pre-existing dormant cells in the tumor and radiotherapy promote their selection, or the radiotherapy itself induces them, their transition to a dormant state in a subpopulation of cancer cells still needs to be validated. 

Dormant quiescence and senescence can aid distinct phenotypes capable of driving tumor relapse [33]. It is believed that persistent or untargeted slow-cycling quiescent cancer cells can become more aggressive and lead to worse prognoses, according to many studies [34,35,36,37,38,39]. This complexity reinforces the necessity to study and explain better the conditions and underlying mechanisms of slow-cycling quiescent and senescent GBM cell formation and their participation in tumor relapse after radiotherapy. 

Recent studies have shown that a linkage between an unjamming transition (UJT) and tumor progression indeed exists [40]. In tumor growth, the uncontrolled proliferation of cancer cells in a confined space generates mechanical compressive stress. IR can provoke human bronchial epithelial cell shape elongation and increased cellular motility, both of which are hallmarks of the UJT, in which epithelial cells move collectively and cooperatively [41]. The bulk of epithelial cells become fluid-like and migratory during morphogenesis, remodeling, or repair, as well as during malignant invasion or metastasis [42,43]. This transition from sedentary to migratory behavior has traditionally been attributed to the epithelial–mesenchymal transition (EMT) or the partial EMT (pEMT) [44]. However, in certain circumstances this transition has been assigned to the recently discovered UJT [45,46,47].

In the case of radiotherapy, GBM cells resistant to IR reportedly display increased DNA damage-response mechanisms, inducing therapy refractivity [48]. Most GBM cells contain different mutations in *TP53* and phosphatase and tensin homolog deleted on chromosome 10 (*PTEN*), the two leading tumor suppressor genes [49]. The variety of mutations and their combinations in individual tumors underlies GBM cell heterogeneity and patient response/outcome to radio- and chemotherapy. Gain-of-function (GOF) *mut-p53* is understudied in GBM. Most studies on p53 in GBM failed to distinguish between *TP53* deletion and GOF mutations [50,51]. In contrast to *p53wt*, GOF *mut-p53* exhibits interactions with distinct transcription factors (e.g., SREBP, VDR, Sp1, ETS2, NFR2, p73, p63) that promote tumor initiation and progression as reviewed elsewhere [52,53,54]. Moreover, *p53mut* inhibition of p63 and p73 promotes tumor cell invasion [55,56]. The *PTEN* gene is induced by p53 [57] and, considering that ΔNp63α can oppose p53 function and bind to p53 responsive elements (REs), it is likely that ΔNp63α may negatively regulate *PTEN* [58]. A wide range of tumorigenic phenotypes are caused by aneuploidy [59,60,61], many of which have been associated with mutant *p53* GOF activities, including, but not limited to: altered proliferation [62], altered metabolism [63], transcriptional reprogramming and chemoresistance [64], migration [65], and invasion and metastasis [66]. Given the strong propensity for cells containing mutant *p53* to become aneuploid and the overlap of tumorigenic phenotypes related to *p53* and *PTEN* alterations, the PGCCs/MGCCs and quiescent slow-cycling formation identified in mutant *p53/PTEN* models must be carefully investigated in connection to corresponding radiation tolerance variation, and ability to shield themselves from IR. 

Hence, to explore the survival and relapse mechanisms in GBM, we used human glioma cell lines differing in radiation tolerance and status of key tumor suppressors as an in vitro model to assess the early stages of GBM response to acute radiation treatment. The present study aimed to investigate whether the PGCCs/MGCCs and the formation of slowly cycling quiescent/senescent cancer cells relate to radiation tolerance (anchorage-independent survival of cell reproductive capacity) in GBM cell lines. We employed the human U-87, LN-229, and U-251 cell lines inheriting decline in radioresistance in that order. These cell lines differ in key tumor suppressors. Both U-87 and U-251 are deficient in *PTEN* activity due to mutant *PTEN* (*PTENmut*), while LN-229 contains wild-type *PTEN* (*PTENwt*). Moreover, U-87 contains wild-type transactivation function-potent *p53* (*TAp53wt*), and U-251 and LN-229 carry mutant (R273H) *p53* GOF (*TAp53mutGOF*) and mutant but transactivation function-potent *p53* (*TAp53mut*), respectively. Between the three GBM cell lines, we found different enrichment in PGCCs/MGCCs and slow-cycling quiescent/senescent populations in response to acute X-ray irradiation at sublethal therapeutic doses of 2–6 Gy. The underlying changes in proliferation, stress-induced premature senescence (SIPS), apoptosis, migration, Ki-67 expression, repopulation propensity, NAD(P)H production, expression of EMT markers, and members of p53 family proteins were investigated. The obtained results may help us better understand the molecular and cellular events underlying an early IR stress-induced adaptation response of GBM cell lines that differ in radioresistance and *TP53/PTEN* status.

## 2. Results

### 2.1. IR Exposure Augments The Fraction of MGCCs and Induces the Metabolic Dormancy of the Bulk of GBM Cells 

**Figure 1:**
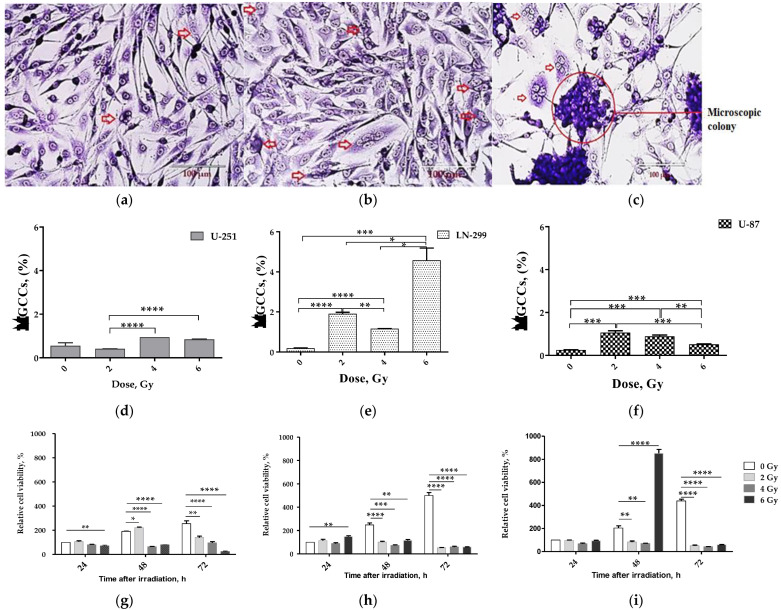
Effect of single-dose irradiation of (**a**,**d**,**g**) U-251, (**b**,**e**,**h**) LN-229, and (**c**,**f**,**i**) U-87 GBM cell lines at doses 2 Gy, 4 Gy, and 6 Gy compared to their control cells. (**a**–**c**) Representative images showing multinucleated giant cancer cells (MGCCs) stained by Wright–Giemsa (MGCCs indicated by red arrows) at 24 h post-IR (cells were seeded in a 96-well plate at a concentration of 100 × 10^3^ cells/0.32 cm^2^). All images were acquired at magnification 20×. (**d**–**f**) The fraction of MGCCs found in the bulk of each GBM cell line at 24 h post-IR. (**g**–**i**) Assessment of the glycolytic NAD(P)H production activity using MTT assay at 24 h, 48 h, and 72 h after IR exposure (cells were seeded in a 96-well plate at a concentration of 2 × 10^3^ cells/0.32 cm^2^). Data are means ± SEM of three independent experiments. * *p* ≤ 0.05, ** *p* ≤ 0.01, *** *p* ≤ 0.001, **** *p* ≤ 0.0001.

Although MGCCs enter a dormancy state, they play an important role in metastasis and display a remarkable resistance to genotoxic stress, including IR [1]. We investigated the plenteousness of MGCCs in GBM cells after 24 h IR exposure using Wright–Giemsa staining (Figure 1).

We found enrichment in MGCC populations of U-87 and LN-229 GBM cell lines in response to acute X-ray irradiation at sublethal therapeutic doses of 2–6 Gy. The U-87 cells and LN-229 cells exposed to any acute single-dose IR demonstrated the most significant (LN-229: 10-, 6-, and 24-fold; U-87: 4-, 3-, and 2-fold, at 2, 4, and 6 Gy, respectively) increase in the proportion of MGCCs (Figure 1e,f), considering that U-251 cells did not differ significantly compared to their control (Figure 1d). The degree of enrichment was highest in LN-229 cells, compared to the modest (but still significant) enrichment in U-87 and the marginal one in U-251 cells. This suggests that transactivation is functional in wild-type or mutated p53 and its driven pathway is necessary and sufficient for PGCC/MGCC formation. 

Finally, we found that some U-87-derived MGCCs could generate numerous small mononuclear cells, previously called Raju cells (or RJ cells) [67] and eventually form cell colonies, a process termed neosis (Figure 1c). Hematoxylin and eosin staining verified the formation of PGCCs/MGCCs and RJ cells 24 h after irradiation of U-87 cells.

At 24 h post-irradiation, the level of glycolytic NAD(P)H production (MTT test) in the bulk populations of transactivation function-potent *p53* cell lines remained either unchanged (U-87) or even modestly increased (LN-229 at 6 Gy), considering that NAD(P)H level with IR dose-dependently declined in the U-251 cell line compared to non-irradiated cells (Figure 1g–i). At 48 h post-IR, only U-87 cells demonstrated a significant (>4-fold at 6 Gy only) increase in NAD(P)H production. A decrease in cellular NAD(P)H was observable in all GBM cell lines at 72 h after irradiation compared to non-irradiated cells irrespective of IR dose.

In sum, these results demonstrate that IR exposure considerably augmented the formation of MGCCs at 24 h with subsequent metabolic dormancy up to 72 h after irradiation.

### 2.2. IR Exposure Triggers Transient Delay in 1D Migration of PTEN^mut^ GBM Cell Lines 

Collective epithelial cellular migration is a requisite event in progression of a carcinoma. IR can provoke human bronchial epithelial cell shape elongation and increased cellular motility, both of which are hallmarks of the unjamming transition (UJT) [41]. Following novel hypothesis, the unjammed phase evolves to accommodate fluid-like migratory dynamics during stages of tissue wound healing but is more energetically expensive than the jammed phase. A metabolic shift toward glycolysis accompanies collective cellular migration characterizing the wound healing process [68]. Hence, our observation of glycolytic NAD(P)H dormancy of the bulk population of GBM cells after irradiation led us to closely examine collective 1D GBM cell migration in a wound healing (“scratch”) test at different times after IR exposure. 

**Figure 2:**
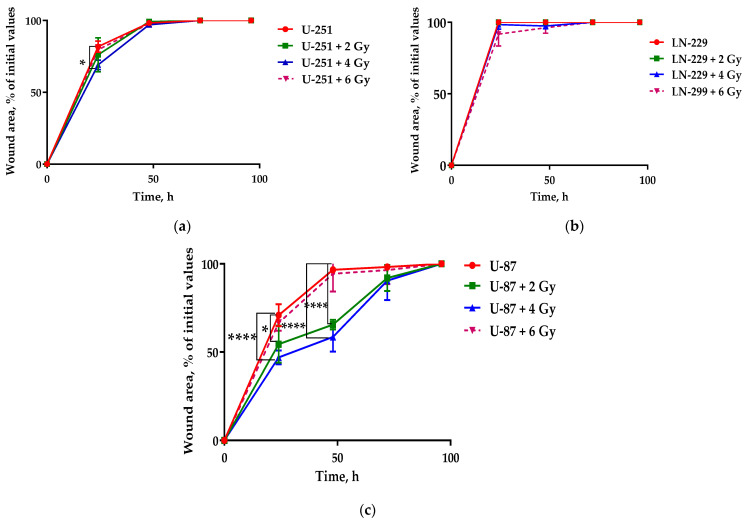
The migration ability of control and treated GBM cells. (**a**) Time-lapse images of a representative scratch assay (wound healing). In each image, the wound area was detected by applying the segmentation algorithm and is plotted in blue. The percentage of the migration ability of (**a**) U-251, (**b**) LN-229, and (**c**) U-87 cells 24 h, 48 h, 72 h, and 96 h after mechanical scratching. Data are means ± SEM of three independent experiments. * p ≤ 0.05, **** p ≤ 0.0001.

As expected from lowering glycolytic NAD(P)H production, IR exposure to 2 Gy and 4 Gy significantly reduced the migration of U-87 cells up to 48 h compared to their control, though expediting their migration by 72 h and reaching control cells’ level by 96 h (Figure 2d). Of note, metabolic dormancy of U-87 cells within 24–48 h (Figure 1i) correlated well with their significant delay in confined 1D migration after 2–4 Gy exposure, while their glycolytic NAD(P)H burst at 6 Gy irradiation might, at least partly, have contributed to recovering migration to the level of non-irradiated cells. An acute single dose of 4 Gy significantly attenuated the 1D migration activity of U-251 cells at 24 h compared to their control, and then it abruptly and oddly reached control cells’ level by 48 h (Figure 2b). Surprisingly, in spite of a significant glycolytic NAD(P)H decline, there were no statistically significant differences in the wound healing migration exhibited by LN-299 cells compared to their corresponding control after IR exposure to any acute single dose (Figure 2c). Thus, in response to IR, only *PTEN*-deficient GBM cells (U-87 and U-251) demonstrated a certain transient delay in migration. The extent and duration of this delay is likely associated with cellular *PTEN* status and glycolytic energy metabolism.

### 2.3. The Impact of IR Stress on Proliferative and Cell Cycling Activity of GMB Cells 

In the present study, the appearance of the MGCCs and metabolic dormancy of the bulk GBM cell population led us to investigate their proliferative vs. non-proliferative features, questioning whether cells left the cell cycle and entered quiescence or stress-induced premature senescence (SIPS) at 24 h after IR exposure. Nuclear Ki-67 and Click-iT EdU^TM^ staining were conducted. Until recently, nuclear Ki-67 staining techniques in clinics operated as if Ki-67 protein levels were a simple on-and-off switch: on during cellular proliferation and off during quiescence and senescence. However, this evaluation seems to be oversimplified. 

**Figure 3:**
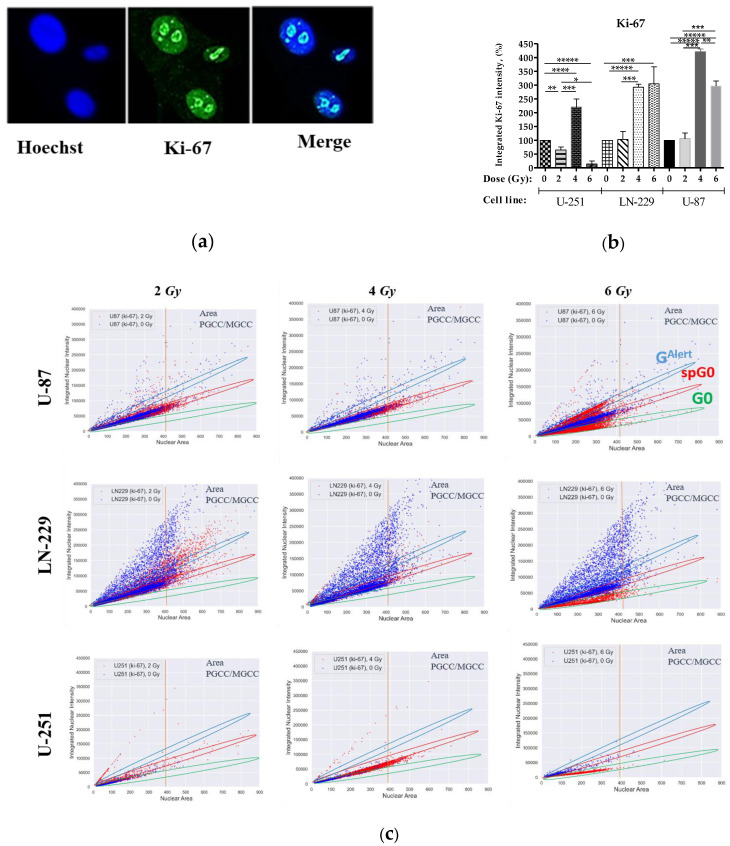
Ki67-coupled fluorescence-intensity analysis. (**a**) Representative immunofluorescence images of cell nuclei (Hoechst 33,342 (blue), Ki-67-stained nuclei (green), and merged images). (**b**) The percentage of Ki-67+ GBM cells in the treated cells was compared with that of the control after normalizing with nuclear counts. (**c**) High-content imaging and analysis of Ki-67-coupled fluorescence of control (blue dots) and irradiated (red dots) GBM cells at 24 h after exposure to different doses of X-rays (cells were seeded in a 96-well plate at a concentration of 2 × 10^2^ cells/0.32 cm^2^). PGCC area denotes the nuclear area threshold to delineate PGCCs/MGCCs of each cell line. The color segmentation of different populations (G^Alert^, spG0 and G0). Data are means ± SEM of more than three independent experiments. * *p* ≤ 0.05, ** p ≤ 0.01, *** p ≤ 0.001, **** p ≤ 0.0001, ***** p ≤ 0.00001.

Indeed, the total fluorescent signal intensities of Ki-67 in all nuclei (Figure 3b) indicated that 4–6 Gy IR caused the most significant increase in the cycling fractions of U-87 (>3–4-fold) and LN-229 (almost 3-fold) cells, while IR exposure at a dose of 4 Gy caused only a 2-fold increase in the same fraction of U-251 cells. 

**Figure 4:**
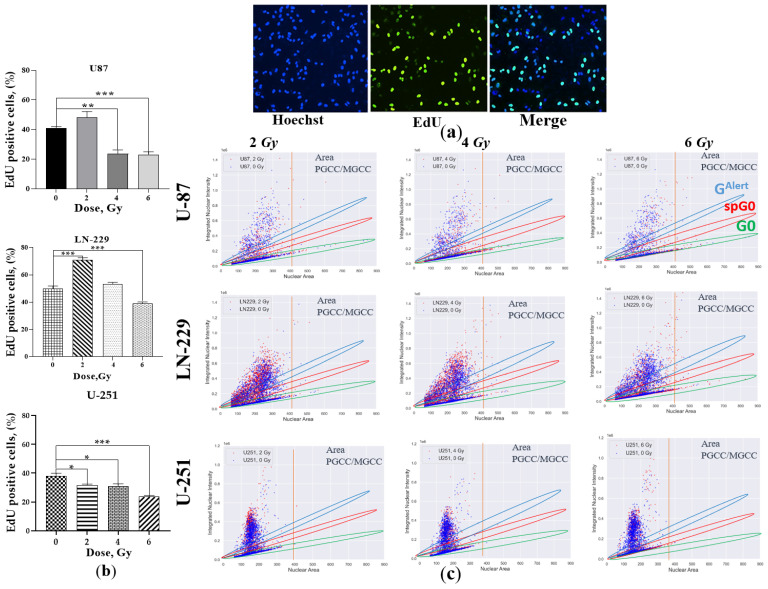
EdU-coupled fluorescence intensity analysis. (**a**) Representative immunofluorescence images of cell nuclei (Hoechst 33,342 (blue), EdU incorporated into DNA (green), and merged images). (**b**) The percentage of EdU-positive GBM cells. (**c**) High-content imaging and analysis of EdU-coupled fluorescence of control (blue dots) and irradiated (red dots) GBM cells at 24 h after exposure to different doses of X-rays rays (cells were seeded in a 96-well plate at a concentration of 2 × 10^3^ cells/0.32 cm^2^). PGCC area denotes the nuclear area threshold to delineate PGCCs/MGCCs of each cell line. The color segmentation of different populations (G^Alert^, spG0 and G0). Data are means ± SEM of three independent experiments. * p ≤ 0.05, ** p ≤ 0.01, *** p ≤ 0.001.

Compared to non-irradiated cells, the lowest IR dose (2 Gy) did not significantly change the fraction of Ki-67+, cycling GBM cells. In contrast, an IR dose of 2 Gy raised the DNA-replicating (dividing) fraction of LN-229 (by 20%) and U-87 (by 10%) cells, while it weakly (by 7%) but significantly (*p* ≤ 0.05) reduced the same fraction of U-251 cells (Figure 4b). The rest of the doses (4 and 6 Gy) significantly reduced dividing cell fractions in all tested cell lines except LN-229 cells at 4 Gy. Collectively, our data point to significantly increased levels of Ki-67 arresting cell proliferation (the fraction of DNA-replicating cells) irrespective of GBM cell line genotype. 

New data demonstrated that careful quantification of Ki-67 antibody staining could reveal more than simply whether a cell is in the proliferative state: it can additionally distinguish a rapidly cycling cell with a very short quiescence from a slowly cycling cell that spends long periods in quiescence before re-entering the cell cycle [69]. 

Hence, we took advantage of using single-cell high-content imaging and analysis to quantify the integrated intensities (IIs) of either Ki-67- or EdU-coupled fluorescent signal per cell nucleus properly. Plotting IIs of either Ki-67 (Figure 3c) or EdU (Figure 4c) staining vs. nuclear area estimated by integrated intensity of Hoechst 33,342 staining per cell allows a single-cell relation of the nuclear area of cancer cells to either their cycling capacity or DNA replication, respectively. Following direct correlation of human cell ploidy and nuclear size [70], the characteristic of PGCCs/MGCCs as the tumor cells with nuclei at least three times the size of the nuclei of diploid cells was proposed [12]. Hence, we first estimated the PGCC threshold (denoted as “PGCC area” in Figure 3c and Figure 4c) of the nuclear area of U-251, LN-229, and U-87 cells as >380 µm^2^, >427 µm^2^, and >406 µm^2^, respectively. Such threshold values enabled analysis of cycling and division capabilities of PGCCs/MGCCs separately from the bulk of GBM cells.

Non-irradiated *TAp53* GBM cell lines (U-87 and LN-229) consist of two populations (blue dots in Figure 3c and Figure 4c). One has nuclear area values directly correlated with either Ki-67 or EdU IIs limited to certain high levels and the other has a scattered distribution of higher Ki-67 and EdU IIs of cells with different nuclear area values. The Ki-67^low^ cells in the first population demonstrated the lowest levels of EdU (EdU^low^) incorporation (Figure 4c), thus resembling the cell population in “spontaneous G0” or spontaneous quiescent state of the cell cycle [71]. Spontaneous quiescent (denoted as “spG0” in Figure 3c) state differs from the classical deep quiescent G0 state in that spG0 cells did not possess distinctively low levels of Ki-67, as would be expected if Ki-67 was “off” in deep quiescence forced by different stress conditions [69]. Most diploid *PTEN*-deficient *TAp53wt* cells (U-87) are in spG0 state with smaller populations of diverse faster cycling and proliferating (Ki-67^high^/EdU^high^) cells representing the second population. The *PTENwt TAp53mut* (LN-229) cells of the same ploidy are almost equally distributed between these two populations. The PGCC/MGCC distribution of non-irradiated *TAp53* GBM cell lines was the opposite, with few demonstrating DNA replication (Figure 4c). Indeed, PGCCs/MGCCs are equally distributed between spG0 and diverse faster cycling (Ki-67^high^) populations of U-87 cells. In LN-229 cells, most PGCCs/MGCCs are in the latter, Ki-67^high^ populations, and with a minor fraction of spG0 cells (Figure 3c). Interestingly, non-irradiated *PTEN-*deficient GOF*p53mut* GBM cells (U-251) demonstrated the overall lowest fraction of cycling (Ki-67+) cells; almost all are diploid and in the spG0 state. In addition, the most significant fraction of U-251 cells were the diverse population of diploid DNA-replicating cells, while there were only a few PGCCs/MGCCs, and they were in spG0 state. Thus, non-irradiated cancer diploid cells and pre-existing PGCCs/MGCCs often spontaneously enter slow-cycling spG0 states of varying duration and depth, which may underlie tumor dormancy and issues with cancer recurrence.

Quiescence causes a diversified state [72]. Among all Ki-67+ GBM cells, X-ray exposure dose-dependently reduced IIs of Ki-67 staining (red dots in Figure 3c), revealing the appearance of the subpopulation with low levels of Ki-67 expression (below the levels characteristic of spG0 state). This subpopulation was also devoid of EdU incorporation (EdU^-^) (red dots in Figure 4c), thus resembling the feature of classical G0 (deep quiescent or “G0” state denoted in Figure 3c and Figure 4c). Of note, G0 populations of all three GBM cell lines were enriched with diploid cells and PGCCs/MGCCs at a dose of 6 Gy only, while at both lower doses, no cells in the G0 population were observed (Figure 3c). For the first time, we demonstrated the advent of another distinct population having Ki-67 expression higher than the level of spG0 cells (Figure 3c) in response to the highest dose X-ray exposure of radioresistant *PTEN*-deficient *TAp53wt* (U-87) cells. The appearance of these two subpopulations manifesting the bifurcation of Ki-67 expression is thought to be a consequence of the proliferation–quiescence decision described earlier [69]. The two major dormant cell populations have features of classical deep quiescence (G0) and so-called “quiescence alert” (G^Alert^), the borderline state between deep quiescence and activation, more akin to quiescence, and re-enter cell cycle more quickly than both deep and spontaneous quiescent cells as described for stem cells previously [73,74]. The 2 Gy irradiation demonstrated enrichment of the spG0 population with both diploid cells and PGCCs/MGCCs, followed by a more pronounced one at the 4 Gy dose. In response to the highest (6 Gy) dose X-ray irradiation of U-87 cells (Figure 3c), most of the diploid cells were equally distributed between G^Alert^ (moderate Ki-67 expression) and G0 (deficient Ki-67 expression) populations. In contrast, the PGCCs/MGCCs enriched all three subpopulations (G^Alert^/spG0/G0) at the same time and treatment dose. Another *PTEN*-deficient, but *TAp53mutGOF*, cell line (U-251) demonstrated enrichment of only the spG0 population with both diploid cells and PGCCs/MGCCs along with the appearance of a small population of very fast cycling (very high Ki-67) cells at 2 and 4 Gy doses exclusively. At 6 Gy, most diploid cells and very few PGCCs/MGCCs were in the G0 population only. The 2 Gy exposure of the *PTENwt/TAp53mut* (LN-229) cells mainly enriched spG0 and G^Alert^ populations with both diploid cells and PGCCs/MGCCs followed by a more pronounced enrichment of only the spG0 population at the 4 Gy dose. The highest dose exposure of the same cells induced enrichment of only the G0 population with diploid cells and PGCCs/MGCCs. 

In sum, the radioresistant *PTEN*-deficient *TAp53wt* cell line (U-87) augmented three distinct populations of dormant PGCCs/MGCCs resembling the Ki-67- and EdU-coupled expression features of G0, spG0, and G^Alert^ quiescence states in response to the highest dose (6 Gy) irradiation. After the same dose of irradiation, in the most radiosensitive *PTEN*-deficient *TAp53mutGOF* (U-251) and less radiosensitive *PTEN*-potent *TAp53mut* (LN-229) cell lines, the former produced hardly any compared to the significant number of dormant PGCCs/MGCCs resembling G0 quiescent state in LN-229 cells. These data suggest that sublethal dose X-ray irradiation promotes selection of dormant cells. The radioresistance correlated well with the proportion of dormant PGCCs/MGCCs resembling the Ki-67/EdU staining features of G0, spG0, and G^Alert^ states in a quiescence or senescence-like adaptation response to different radiation stress doses. 

### 2.4. Impact of Metabolism and Apoptosis on Anchorage-Independent Growth Efficiency and Radiosensitivity of GBM Cell Lines

The survival of cancer cells after IR stress is indicative of cancer cell fitness and fate in harsh environments. Radiotherapy has been implicated in the change in the biological behavior of GBM [2]. Anchorage-independent growth (AIG) is a hallmark of attachment-regulated apoptosis (anoikis) resistance forming a relapse population and the path to further steps in metastasis. To find out whether X-ray irradiation induces the AIG of GBM cells, we used a soft agar colony formation assay. 

**Figure 5:**
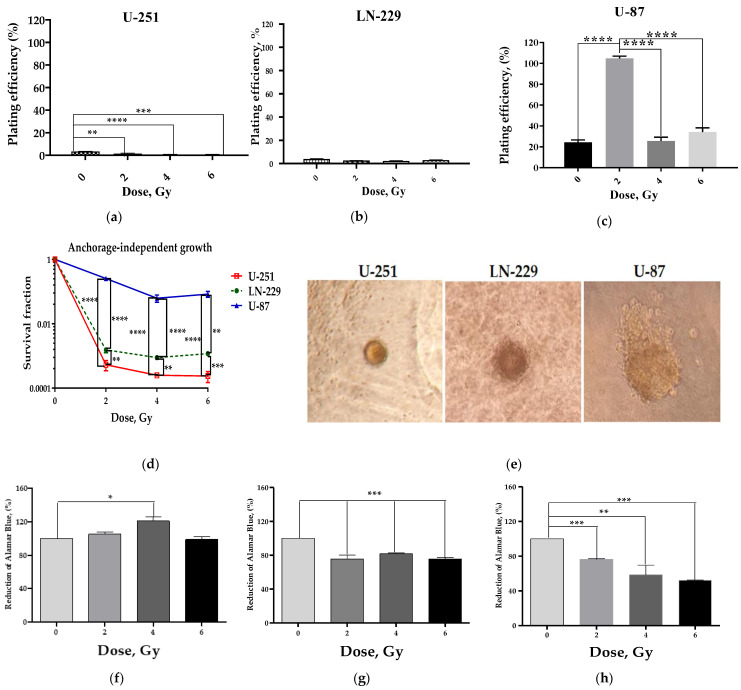
Anchorage-independent growth (soft agar colony formation assay) of (**a**) U-251, (**b**) LN-229, and (**c**) U-87 GBM cells exposed to acute 2 Gy, 4 Gy, and 6 Gy single doses of IR compared to non-irradiated control cells. (**d**) Survival fractions of control and their irradiated GBM lines after 21 days of cultivation after irradiation. (**e**) Representative light microscopic images of the colonies of GBM cell lines acquired at 20× magnification. Assessment of the mitochondrial NAD(P)H production activity of (**f**) U-251, (**g**) LN-229, and (**h**) U-87 cells one week after IR exposure to acute single doses of 2 Gy, 4 Gy, and 6 Gy compared to their control cells using alamarBlue assay (cells were seeded in soft agar in a 96-well plate at a concentration of 24 × 10^2^ cells/0.32 cm^2^). Data are means ± SEM of more than three independent experiments. * p ≤ 0.05, ** p ≤0.01, *** p ≤ 0.001, **** p ≤ 0.0001.

IR exposure of U-87 cells at 2 Gy elicited a significant (5-fold) increase in AIG colonies compared to their non-irradiated control (Figure 5c). X-ray irradiation caused significant dose-related reduction of U-251 cell AIG compared to their non-irradiated control cells (Figure 5a). As shown in Figure 5b, the AIG efficiency of irradiated LN-229 cells and their basal control did not differ significantly after exposure to any acute single-dose irradiation. Dose-dependent analysis of the survival fraction of GBM cell lines confirmed significantly higher development of the relapse population in U-87 cells compared to LN-229 and U-251 cells by the 21st day after irradiation (Figure 5d). Thus, U-87 cells possessed the highest anchorage-independent survival of cell reproductive capacity, confirming their highest overall radioresistance.

Notably, U-251 cells formed more dense colonies, compared to LN-229 and U-87 cells (Figure 5e). Most of the U-87-derived colonies were diffuse with a halo of cells migrating into the agar, indicating the highest invasiveness of cells constituting the colony. 

To investigate whether IR-induced metabolism impairment underlies the clonogenic survival of GBM cell lines, we used a well-established alamarBlue assay in a 3D anchorage-independent format [75,76]. Cells were plated on soft agar in 96-well microplates and allowed to grow for one week post-IR before quantification of the reduction rate of the resazurin into fluorescent product resorufin by metabolically active viable cells. In essence, alamarBlue reduction may signify an impairment of cellular metabolism, albeit it is not necessarily specific to interruption of electron transport and mitochondrial dysfunction [77].

IR exposure increased alamarBlue reduction by U-251 cells, reaching significance compared to control at a dose of 4 Gy only (Figure 5f). Compared to the non-irradiated control, colonies of LN-229 cells showed a dose-independent decrease in metabolic activity after IR exposure (Figure 5g). The metabolic activity of U-87 cell colonies was dose-dependently reduced compared to control at one week after IR exposure (Figure 5h). Together, these data indicated that metabolic dormancy seems to be characteristic of the highest clonogenic survival and fitness underlying the radioresistance of *PTEN*-deficient *TAp53wt* cell line U-87. In contrast, the metabolic silence was not beneficial for the clonogenic survival and fitness of *PTEN*-competent *TAp53mut* LN-229 cell progeny (Figure 5b) after irradiation despite the earliest highest increase in their MGCCs (Figure 1e).

To better understand this apparent discrepancy, we performed analysis of apoptosis in the bulk of GBM cells 24 h after irradiation using YO-PRO-1 staining. YO-PRO-1 is a green fluorescent DNA dye which serves as a marker for cells with a compromised plasma membrane, and as a potential early marker of cell death [78]. 

**Figure 6:**
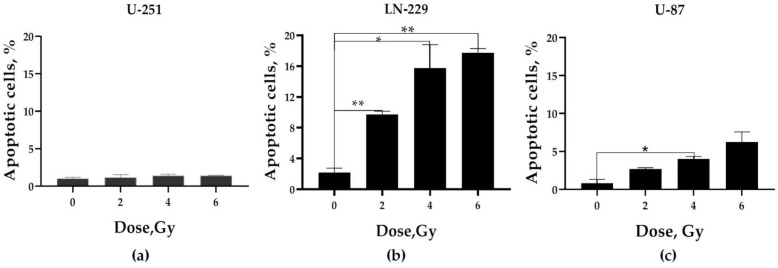
Assessment of apoptosis by YO-PRO-1 in GBM cell lines. Percentages of apoptotic U-251 (**a**), LN-229 (**b**), and U-87 (**c**) cells demonstrate highest rate of apoptosis (YO-PRO-1 positive) in LN-229 cells compared to parental cells 24 h after exposure to IR. Data are means ± SE. * *p* ≤ 0.05, ** *p* ≤ 0.01.

As expected, the presence of functional *TAp53* dose-dependently increased the proportion of apoptotic cells both in U-87 and LN-229 lines after exposure to 2–6 Gy (Figure 6b,c) compared to non-irradiated cells. 

Remarkably, compared to the low increase in apoptotic *PTEN*-deficient U-87 cells, the *PTEN*-competent LN-229 cells (Figure 6b) showed the most prominent increase (5–9-fold at 2–6 Gy) in the proportion of apoptotic cells after any acute single-dose IR exposure. In contrast, *PTEN*-deficient *TAp53mut GOF* cells (U-251) demonstrated no change in the proportion of apoptotic cells after any dose exposure compared to non-irradiated cells. These data suggested that, albeit functional *p53* alone sensitizes both cell lines to IR-induced apoptosis, the engagement of functional *PTEN*, another crucial tumor suppressor, might elicit a much more prominent apoptotic response attenuating the clonogenic survival and fitness of LN-229 progeny even after the earliest massive MGCC induction by acute single doses of IR exposure. Nonetheless, we cannot exclude that other modes of cellular DNA damage response (such as mitotic catastrophe or senescence) might be engaged when responding to single acute doses of X-rays in GBM cell lines.

### 2.5. The Impact of X-Ray Irradiation on Stress-Induced Premature Senescence (SIPS) of GMB Cells

A cell must definitively leave the cell cycle to become dormant, either quiescent or senescent. Our observed Ki-67 staining bifurcation pattern may indicate that, soon after noxious stimulus, damaged cells leave the cell cycle. We proposed that those that successfully recover from damage re-enter the cell cycle, while cells with irreparable damage become senescent. Senescent cells are also Ki-67 negative or weakly express Ki-67, mimicking cells residing in the G0 state [79]. The increased level of SA-β-Gal reactivity is a prominent marker of high lysosomal activity and lysosomal content during the stress response. Although the level of SA-β-Gal was observed in both senescence and quiescence states, it was higher in senescence [80]. Hence, we analyzed SA-β-Gal reactivity in response to irradiation of GBM cells at 24 h after the IR-induced stress. 

**Figure 7:**
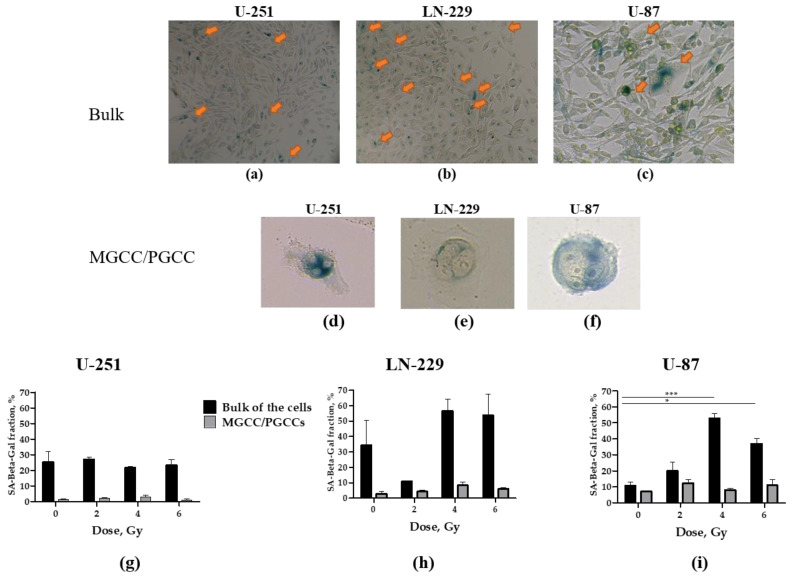
Senescence-associated β-Gal staining of GBM cells and their corresponding MGCCs/PGCCs 24 h after X-ray irradiation. Representative light microscopy staining images of SA-β-Gal cytochemistry showing SA-β-Gal positive cells (blue stain) at both the bulk GBM populations (×20) (**a**–**c**) and individual MGCC/PGCC (×40) (**d**–**f**). Orange arrows indicate SA-β-Gal + cells. The percentage of SA-β-Gal + cells in the same populations of (**g**) U-251, (**h**) LN-229, and (**i**) U-87 cells seeded at a concentration of 5 × 10^3^ cells/0.32 cm^2^. * *p* ≤ 0.05, *** *p* ≤ 0.001.

p53 is known to play a critical role in cellular senescence [81]. Among non-irradiated GBM cells, *TAp53wt* U-87 cells had the lowest and the highest SA-β-Gal + fractions in the bulk (10%) and in PGCC/MGCC (5%) populations, respectively (Figure 7i). Compared to U-87 cells, the bulk and PGCC/MGCC populations of both cell lines with mutated *TAp53* (LN-229 and U-251) consist of almost equally higher (32% and 25%) and lower (2% and 1%) SA-β-Gal + fractions, respectively (Figure 7g–h).

In the bulk population of U-87 cells, IR dose-dependently augments the SA-β-Gal + fraction up to 50% at 4 Gy, albeit declining to 35% at 6 Gy (Figure 7i). In the bulk population of LN-229 cells, IR significantly decreased the SA-β-Gal + fraction by 6-fold at 2 Gy, considering that this fraction increased up to 53% at 4 and 6 Gy, albeit it did not reach statistical significance over non-irradiated cells (Figure 7h). 

In the PGCC/MGCC population of U-87 cells, the SA-β-Gal + fraction increased two-fold to a maximal level 10% at 2 Gy, then decreased to 5% and 7% at 4 and 6 Gy, respectively (Figure 7i). No IR dose significantly changed the SA-β-Gal + fractions in PGCC/MGCC populations of LN-229 nor U-251 cells. Moreover, the SA-β-Gal + fraction in the bulk population of U-251 was not affected either (Figure 7g). Collectively, these data show a direct correlation between the radioresistance (the anchorage-independent survival of cell reproductive capacity and development of the relapse population) of U-87 cells and the degree of SIPS (fraction of SA-β-Gal +/Ki-67^low^ cells) in the PGCC/MGCC population.

### 2.6. Molecular Signatures of EMT and p53 Family Proteins of GBM cell Lines in Response to IR Stress

The EMT mechanism cannot only make PGCCs manifest the relevant features of tumor stem cells but can also be part of the cause of the metastasis of tumor cells, thereby linking stemness and metastasis in PGCCs [82,83]. Overexpression of FRA-1 (encoded by the *FOSL1* gene), a leucine zipper protein forming the transcription factor complex AP-1, can promote tumor dissemination due to its possible role in EMT-like processes, and metastatic spreading, by driving the expression of EMT-inducing transcription factors, cytokines, and microRNAs [3,4,18,84]. Hence, we tested FRA-1 expression levels after exposure to different doses of X-ray irradiation. 

**Figure 8:**
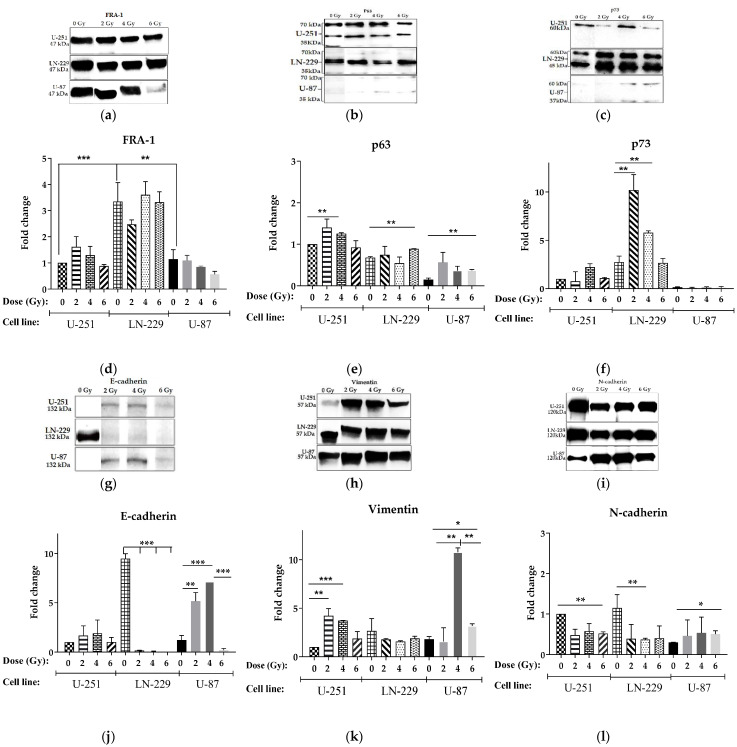
Semi-quantitative Western blot analysis of expression of EMT-related markers in control (non-irradiated, 0 Gy) cells vs. irradiated GBM cells 24 h after exposure to different doses of X-rays. Western blot of (**a**) FRA-1 protein, (**b**) p63, (**c**) p73, (**g**) E-cadherin, (**h**) vimentin, and (**i**) N-cadherin and quantification of (**d**) FRA-1 protein, (**e**) p63, (**f**) p73, (**j**) E-cadherin, (**k**) vimentin, and (**l**) N-cadherin expression. Data are means ± SEM of more than three independent experiments. * *p* ≤ 0.05, ** *p* ≤ 0.01, *** *p* ≤ 0.001.

Compared to both non-irradiated *PTENmut* cell lines, non-irradiated *PTENwt* LN-229 cells showed significantly (three-fold, *p* < 0.05) higher FRA-1 expression (Figure 8d). Notably, the exposure to any acute single-dose IR induced a dose-dependent, albeit insignificant, decline in the FRA-1 expression in both of the *PTENmut* cell lines compared to corresponding control cells (Figure 8d). In contrast, FRA-1 expression of LN-229 (*PTENwt*) cells was not changed in response to any IR dose. Ergo, we propose that IR exposure did not substantially affect the FRA-1 expression in irradiated compared to non-irradiated GBM cells irrespectively of their *PTEN* status. 

P73 and p63, p53 protein family members, are structurally and functionally homologous to p53 [85], and they have been shown to regain the tumor suppressive functions of p53 in *p53mut*’s absence. Notwithstanding, like *p53wt*, this activity is inhibited by protein–protein interactions between *p53mut* and p63/p73. As anticipated, the *p53wt* U-87 cells expressed the lowest basal (non-irradiated) levels of p63 and p73 (Figure 8e–f). Among mut-*p53* cell lines, LN-229 and U-251 cells expressed the highest basal levels of p73 and p63, respectively. Despite carrying GOF *p53mut*, U-251 cells exposed to 4 Gy expressed about 1.5- and 2.2-fold higher p63 and p73, respectively, compared to their basal levels (Figure 8e–f). Moreover, 6 Gy exposure of LN-229 and U-87 cells carrying *TAp53* resulted in a significant (1.3- and 2.6-fold, respectively) increase in p63 expression compared to their corresponding basal levels. Of note, both *PTENmut* cell lines (U-251 and U-87) demonstrated a similar pattern (albeit to a different extent) of dose-related p63 and p73 expression changes in response to IR exposure (Figure 8e–f). In contrast, 2 Gy and 4 Gy exposure of *PTENwt* LN-229 cells significantly augmented (3.76-, 2.11-fold, respectively) only the p73 expression level compared to the basal level, albeit it returned to the basal level after exposure to 6 Gy (Figure 8f). These data proposed that the innate *PTENmut* status of GBM cells might jeopardize IR stress-induced p63/p73 expression.

The finding of these biomarkers being highly upregulated in GBM cells aroused our interest in the association of their expression with the development of EMT traits promoting GBM cell migration after IR exposure. Recent data demonstrated that *PTEN* inactivation contributes to EMT in lung cancer cells [86], and the restoration of *PTENwt* expression converted breast cancer cells with mesenchymal traits to an epithelial phenotype and inhibited cancer stem cell (CSC) activity [87]. Vimentin and N-cadherin could support cellular integrity and act in several cell signal pathways to modulate the motility and invasion of cancer cells [88]. It is still not clear whether p53 directly regulates the gene encoding cadherin. Hence, we assessed the protein expression patterns of E-cadherin, N-cadherin, and vimentin, as the classic EMT markers in human GBM, using Western blot analysis. 

As anticipated, the basal levels of epithelial E-cadherin expression were almost 10-fold higher in *PTENwt* LN-229 compared to *PTENmut* cells (both U-251 and U-87) (Figure 8j). At the same time, the basal expression of both vimentin and N-cadherin, mesenchymal traits, was almost the same in *PTENwt* LN-229 and both *PTENmut* cells (Figure 8k–l).

In *PTEN*-deficient cell lines, IR-induced genotoxic stress generates a mixed EMT phenotype. Exposure of *p53wt* U-87 cells dramatically dose-dependently augmented E-cadherin (4.3- and 5.9-fold at 2 Gy and 4 Gy, respectively) (Figure 8j). Among the mesenchymal traits, vimentin was upregulated (by 5- and 1.8-fold at 4 Gy and 6 Gy, respectively) (Figure 8k), while N-cadherin demonstrated only a subtle increasing trend compared to control (non-irradiated) U-87 cells (Figure 8l). The vimentin expression was 4.2- and 3.8-fold upregulated in GOF *p53mut* U-251 cells exposed to 2 Gy and 4 Gy, respectively (Figure 8k) compared to their control cells. N-cadherin was downregulated in U-251 cells exposed to any dose compared to control (Figure 8l). 

Strikingly, any dose of IR exposure of *PTENwt/p53mut* LN-229 cells significantly downregulated expression of both cadherins (more than 63- and 3-fold for E- and N-cadherin, respectively) without a statistically significant effect on vimentin expression (Figure 8j–l). Thus, the absence of *PTEN* in *p53wt* GBM cell line U-87 is likely associated with the upregulation of p63 along with both epithelial (E-cadherin) and mesenchymal markers (N-cadherins and vimentin) as a possible molecular fingerprint of resistance to acute single-dose IR exposure.

## 3. Discussion

Past decades of glioma research demonstrated a central role of p53 in the regulatory network of gliomagenesis and that the *p53* status is closely associated with the disease progression and survival of patients with GBM during radio- and chemotherapy [89,90]. In the present study, we examined the impact of X-ray radiation exposure on the molecular and cellular IR stress-induced adaptation response of three conventional GBM cell lines: U-251 (*TAp53mut* GOF), LN-229 (*TAp53mut*), and innately mostly resistant to radiation U-87 (*TAp53wt*). Our data indicate that IR differentially affects stress-induced adaptation response associated with the applied single dose and *p53/PTEN* status. 

First, we demonstrated MGCCs were more abundant in LN-229 and U-87 cells compared to their corresponding controls after IR exposure at any single dose, endowing the capacity of cancer recurrence and the creation of a stem cell-like progeny [23,24,25]. Furthermore, our data are more comprehensive and enrich the previously published data by Kaur et al. [17], who explored therapeutically irrelevant lethal doses (> 8 Gy) of IR exposure of U-87 and SF268 GBM cell lines in establishing a recurrent in vitro model. Differing from that study, we used therapeutically relevant doses (2–6 Gy) to investigate early and delayed cellular and molecular IR stress-induced adaptation reactions in GBM cell lines with different innate resistance to radiation. At 24 h after IR, we observed the augmented formation of MGCCs in the U-87 line, eventually generating numerous colonies of small mononuclear cells resembling features of cells previously called RJ cells, a process termed neosis [67]. If the MGCC formation is a consequence of de novo radiation-induced homotypic cell fusions of the innately radioresistant U-87 cells, as proposed by Kaur et al., then the low IR doses inducing such a process were much more effective in *p53* transactivation-potent lines U-87 and LN-229 compared to *GOFp53mut* (U-251) cells (Figure 1d–f). Such an early survival response (by neosis) was also rather unexpected because the other data show that the stemness may be attained after genotoxic stress in induced PGCCs of ovarian and lymphoblastoid cancer cells much later—around day 5 after irradiation [12,91]. Moreover, the main proportion of induced polyploid cells undergo apoptotic crisis by day 5 and only a much smaller fraction shows a survival response (by neosis) from day 7 on. In this regard, we did not investigate polyploidy and stemness acquisition in our present study. Notwithstanding, such an observed early stress-induced response correlates quite well with time of appearance of “dormancy-related” features, including polyploidy, in CSC-like (CD44+/CD133+) vs. non-CSC (CD44-/CD133-) isogenic subpopulations of A549 and H1299 non-small lung cancer cells (NSCLCs) as an early marker (predictor) of their behavior after the same type of genotoxic stress [92]. Moreover, the behavior and dynamics of each feature’s appearance in lymphoblast [12,91] and solid (NSCLCs or GBM studied by us) tumors are supposed to be different. In the present study, the degree of MGCC enrichment was highest in LN-229 cells, compared to the modest (but still significant) enrichment in U-87 and the marginal one in U-251 cells (Figure 1d–f), suggesting that transactivation is functional either in wild-type or mutated *p53* and its driven pathway might be associated with MGCC formation efficacy. This observation brings more support to our preceding hypothesis that under genotoxic and nutrient stress, such as IR exposure and serum starvation, functional *p53* may promote entering a safer, presumably “dormant” state to preserve solid tumor cells from death irrespectively of their stemness [92]. 

In the study by Kaur et al., with lethal doses of IR initially for 48h, there was rapid proliferation of GBM cell lines, which was followed by massive cell death. Here, we added new and more comprehensive data on the early stress-induced proliferative response of GBM cell lines. Indeed, the fraction of DNA-replicating cells can be modulated by each therapeutically relevant dose of IR below 8 Gy (Figure 4b). Moreover, our data suggest that the significantly increased levels of Ki-67 (Figure 3b) attenuate cell proliferation (the fraction of DNA-replicating cells) (Figure 4b) irrespectively of GBM cell line genotype, thus corroborating the same observations on other tumors [93]. In addition, our data suggest that sublethal dose X-ray irradiation promotes selection of dormant cells, and the radioresistance correlated well with the proportion of dormant PGCCs/MGCCs resembling the Ki-67/EdU staining features of G0, spG0, and G^Alert^ states in quiescence- and/or senescence-like adaptation response to different radiation doses.

Our data do not confirm the belief that all MGCCs seen in GBM cell lines are induced by radiation, but are not the pre-existing giant cells from a parent population, as was outlined by Kaur and co-workers. Additionally, we do not confirm their data that the β-galactosidase staining showed positivity only in MGCCs, rather it was even higher in the bulk population of mononucleated cells (Figure 7g–i), as seen in other tumors as well. The possible source of this discrepancy might lie in how the MGCC population was isolated and investigated in that study. Indeed, Kaur and co-workers used fluorescence-activated cell sorting-based side and forward scatter identification of giant cytoplasm cell populations, which obviously include both mononucleated tumor cells with extremely large cytoplasm (or cell aggregates) and MGCCs. In contrast, we explore single-cell high-content imaging and analyses of the size of the nuclear area (estimated by integrated intensity of Hoechst 33,342 staining per cell) which enable evaluation of the IR effect on mononucleated cells vs. PGCC/MGCC populations separately. 

Notwithstanding, it is not solely the total β-Gal positivity and the amount of MGCCs that cause the fate of irradiated LN-229 cell lines, as follows from the low yield of relapse cell progeny (Figure 5b), despite the initially highest MGCC formation (Figure 1e). Our apoptosis data shed light to resolve this issue. According to an earlier report [94], our data indicated that, although functional *p53* alone sensitizes both U-87 and LN-229 cell lines to IR-induced apoptosis (Figure 6b,c), *PTEN* engagement might elicit a much more prominent apoptotic response (Figure 6b). That can attenuate the overall clonogenic survival and fitness of LN-229 progeny even after preceding massive MGCC induction by acute single-dose IR exposures. Nonetheless, we could not exclude the incapability of anastasis, an apoptosis reversal [95] by LN-229 cells. Though we did not rule out that MGCCs are predominant contributors to the relapse cell population postulated by Kaur et al., we certainly make clear that the cell *PTEN/TP53* genotype can be more pivotal for anchorage-independent survival of cell reproductive capacity (Figure 5a–c) under low-dose IR-induced genotoxic stress. 

Cellular unjamming is the collective fluidization of cell motion and has been linked to many biological processes, including development, wound repair, and tumor growth. UJT and pEMT have certain common aspects of collective cellular migration, although the extent to which these processes are distinct, overlapping, or perhaps even the same has remained unclear. Metastases often retain E-cadherin expression, and EMT is not required for metastasis arising from clusters of tumor cells [96]. We found that IR stress triggers the delay of confined GBM cell migration after exposure in transitions correlated with *PTEN/p53* status and cell shape, leading us to examine the contributions of cell–cell adhesion and anchorage-independent colony growth in unjamming transitions after IR-induced stress. 

We found almost 10-fold higher basal E-cadherin expression in *PTENwt* LN-229 compared to *PTENmut* cells (both U-251 and U-87) (Figure 8j), which might underlie the highest confined migration rates, suggesting higher basal (non-irradiated) UJT of these cells (Figure 2c). Unexpectedly, the expression of both vimentin and N-cadherin, the basal mesenchymal traits, was also higher in *PTENwt* LN-229 compared to both *PTENmut* GBM cells (Figure 8k,l).

In *PTEN-*deficient cell lines, IR genotoxic stress generates cells with a hybrid epithelial/mesenchymal (E/M) phenotype, that may possess higher tumor initiation and metastatic potential as compared to cells on either end of the EMT spectrum [97]. In our study, IR exposure of *p53wt* U-87 cells dramatically dose-dependently augmented E-cadherin (4. 3- and 5.9-fold at 2 Gy and 4 Gy, respectively) (Figure 8j), while inducing only a subtle rise in E-cadherin in GOF *mut-p53* U-251 cells. Vimentin expression was upregulated by 5- and 1.8-fold at 4 Gy and 6 Gy, respectively (Figure 8k), albeit with a subtle increasing trend in N-cadherin expression compared to control (non-irradiated) U-87 cells (Figure 8l). Of note, these changes were well correlated with the extent of the transient delay of collective migration of respective cell lines (Figure 2b,c). Altogether, the increase in E-cadherin and, to a lesser extent, vimentin may be attributed to the dose-dependent delays of confined migration as an indicator of IR-induced transient reduction of UJT in *PTEN*-deficient cell lines. On the other hand, significant downregulation of E-cadherin at the 6 Gy dose was accompanied by the rescue of the migration level of non-irradiated U-87 cells (Figure 2c). Strikingly, any dose of IR exposure of *PTENwt/p53mut* LN-229 cells significantly downregulated expression of both cadherins (more than 63- and 3-fold for E- and N-cadherin, respectively) without a statistically significant effect on vimentin expression (Figure 8j,k,l). A major consequence of E-cadherin downregulation is the loss of stable epithelial cell–cell adhesive junctions, apico-basal cell polarity, and epithelial tissue structure, thereby facilitating the release of cancer cells from the primary tumor site [98,99]. Thus, the IR stress-induced downregulation of E-cadherin is likely a prerequisite to support the basal (non-irradiated) level of migration of *PTENwt/p53mut* LN-229 and *PTEN*-deficient/*p53wt* U-87 cells. 

In this regard, our data may be contradictory to the recent data on increased E-cadherin as a strong indicator and prerequisite of compressive stress-induced UJT and increased migration rate in “scratch” tests of metastatic mouse breast cancer cell line 4T1 [100]. The authors concluded that the upregulation of E-cadherin is required for compression-induced unjamming, and that E-cadherin-dependent cell–cell adhesion is a key regulator and effector upon compression. The causes of these discrepancies might be as follows. First, we explored IR (genotoxic) stress-induced UJT; second, different cell origin, i.e., GBM in our case vs. mammary tumors; and, lastly, we did not assess the E-cadherin expression after 96 h post-IR, by which time cells return to the level of basal (non-irradiated) migration. In general, our data support the belief that E-cadherin may play a pivotal role in regulation of stress-induced UJT. In contrast to compressive stress, the upregulation of both epithelial (E-cadherin) and mesenchymal markers (N-cadherins and vimentin) is strongly associated with transient attenuation of IR stress-induced UJT in *PTEN*-deficient *p53wt* GBM cells (U-87).

Next, we investigated whether the cadherin-mediated cell–cell adhesion and IR stress-induced attenuated UJT may cause cells to leave the cell cycle and enter quiescence or stress-induced premature senescence (SIPS) at 24 h after IR exposure. For these purposes, nuclear Ki-67 and Click-iT EdU^TM^ staining were conducted as complementary cell proliferation assays. We found that the fraction of Ki-67+ GBM cells (Figure 3) did not correlate with the proportion of DNA-replicating (dividing) cells, suggesting that the proportion of Ki-67+ cells does not mirror the cell proliferation capability. In support of this, it was genetically shown that Ki-67 is not required for cell proliferation in tumors, albeit it is required for all stages of carcinogenesis [93]. Ki-67 is an essential mediator of ectopic heterochromatin formation and links heterochromatin organization to cell proliferation. Heterochromatin reorganization caused by Ki-67 downregulation does not interfere with cell cycle progression or cell proliferation, but likely contributes to remodeling of gene expression [101].

Using single-cell high-content imaging and analysis, we quantified the integrated intensities (IIs) of either Ki-67- or EdU-coupled fluorescent signal per cell nucleus. Plotting IIs of either Ki-67 (Figure 3c) or EdU (Figure 4c) staining vs. nuclear area estimated by Hoechst 33,342 staining allowed a single-cell relation of the nuclear area of cancer cells to either their cycling capability or DNA replication, respectively. We make it clear that non-irradiated diploid GBM cells and their pre-existing PGCCs/MGCCs often spontaneously enter a slow-cycling state resembling a spontaneous quiescent (denoted as “spG0” in Figure 3c) state, which may underlie tumor dormancy and issues with cancer recurrence. The spG0 state differed from the classical deep quiescent G0 state in that spG0 cells did not possess distinctively low levels of Ki-67, as would be expected if Ki-67 was “off” in deep quiescence forced by different stress conditions [69].

For the first time, we demonstrated the advent of another distinct population having Ki-67 expression higher than the level of spG0 cells (Figure 3c) in response to the 6 Gy dose X-ray exposure of innately radioresistant U-87 cells. Intriguingly, this advent is strongly associated with both confined migration, recovering to the basal level characteristic of non-stressed cells, and downregulation of E-cadherin (Figure 8j). With manifestation of the proliferation–quiescence decision described earlier [69], such a bifurcation of Ki-67 expression points to the appearance of the two major dormant cell populations with features of classical deep quiescent (G0) and so-called “quiescence alert” (G^Alert^) states, re-entering the cell cycle more quickly than both G0 and spG0 quiescent cells as described for stem cells previously [73,74]. IR stress-induced attenuated UJT (Figure 2d) by upregulated E-cadherin expression (Figure 8j) at 2 Gy was accompanied by enrichment of the spG0 population with both diploid cells and PGCCs/MGCCs, followed by a more pronounced enrichment at the 4 Gy dose. Rescue of IR stress-induced attenuated UJT by downregulation of E-cadherin expression (Figure 8j) was complemented by equal distribution of most diploid cells between G^Alert^ (moderate Ki-67 expression) and G0 (deficient Ki-67 expression) populations in response to the highest (6 Gy) dose X-ray irradiation of U-87 cells. In contrast, the PGCCs/MGCCs enriched all three subpopulations (GAlert/spG0/G0) at the same time and with the same dose (6 Gy). Our data suggest that the radioresistance is correlated well with the proportion of dormant PGCCs/MGCCs with the Ki-67/EdU staining features of G0, spG0, and G^Alert^ states in a quiescence-like response to different radiation stress doses. In addition, IR stress-induced modulation of E-cadherin expression and the selection of U-87 dormant cells likely transiently regulate their UJT. 

We proposed that those that successfully recover from IR stress damage re-enter the cell cycle, while cells with irreparable damage become senescent. Senescent cells are also Ki-67 negative or weakly express Ki-67, mimicking cells residing in the G0 state [79]. Differing from quiescent cells, senescent cells can be identified based on their high senescence-associated β-galactosidase (SA-β-Gal) activity, presence of p16, and degradation of MDM2 [102]. To delineate the impact of senescence on radioresistance, we analyzed SA-β-Gal reactivity in response to irradiation of GBM cells at 24 h post-IR-induced stress. Our data propose a direct correlation between the radioresistance (the anchorage-independent survival of cell reproductive capacity and development of the relapse population) and the degree of SIPS traits (fraction of SA-β-Gal +/Ki-67^low^ PGCCs/MGCCs, lowering of bulk cell metabolism, apoptosis, and DNA-replicating cells) in the U-87 cell line.

To examine the contributions of E-cadherin-mediated modulation in unjamming transitions in anchorage-independent colony growth after IR-induced stress, we analyzed anchorage-independent growth (AIG) as a hallmark of attachment-regulated apoptosis (anoikis) resistance and the path to further steps in metastasis using a colony formation assay in soft agar and 3D culture alamarBlue assay of mitochondrial metabolism impairment. Our data indicated that significant IR-induced attenuation of UJT of U-87 cells correlated with the highest and most significant (5-fold) increase in anchorage-independent growing colonies compared to their non-irradiated control and other GBM cell lines (Figure 5a–c). These colonies were the largest with a halo of migrating cells in soft agar (Figure 5e), having significant mitochondrial dysfunction (Figure 5h). We propose that IR-induced attenuation of UJT and subsequent metabolic dormancy seem to be characteristic of the highest clonogenic survival and fitness underlying the highest radioresistance (Figure 5d) of *PTEN*-deficient *p53wt* cell line U-87. 

Our effort to define the molecular signatures of IR-induced modulation of UJT corroborates the recent observations that the EMT/pEMT and UJT are quite different processes [103]. Even the overexpression of FRA-1 can promote tumor dissemination due to its possible role in EMT-like processes and metastatic spreading [3,4,18,84], but it does not likely play a role in tested GBM cells as indicated by unaltered IR-stress-induced FRA-1 expression in irradiated compared to non-irradiated GBM cells irrespectively of their *PTEN* status (Figure 8d). These results suggest a possible role of other transcription factor(s) (such as Twist, Snail, ZEB-1/2) in maintenance of dormancy (quiescence and/or SIPS) and cancer stem-like phenotypes of human GBM cells in response to IR exposure. Alternatively, the phosphorylation of FRA-1, which is the modification required for FRA-1–JunB complex formation, needs to be investigated.

Our data proposed that the native *PTENmut* status of GBM cells might jeopardize IR stress-induced p63/p73 expression. In addition, even small (LN-229) or large (U-87) increases in p63 may be needed to increase the number of MGCCs in the cell line (Figure 8e). On the contrary, the *GOF p53mut* genotype (U-251 cells) likely disabled the same cell enrichment despite the profile of IR dose-dependent p63 induction being much the same as in *p53wt* cells (U-87 cells). Of note, significantly higher basal and IR stress-induced p73 expression (Figure 8f) might underlie the highest migration rate of LN-229 cells (Figure 2b).

*p53mut* proteins boost cancer cell radioresistance by promoting multiple pathways that protect cancer cells from stress stimuli, such as metabolic alterations, DNA damage, oxidative stress, and tumor microenvironment [104]. Given that they share amino acid sequence identity reaching 63% in the DNA-binding domain [105], p63 and p73 are implicated in many biological activities, including cell proliferation, survival, apoptosis, development, differentiation, self-renewal, senescence, and metabolic adaptation regulation triggered in response to oxidative stress [106,107]. Furthermore, *TP63* and *TP73* downregulation may contribute to metastasis and treatment resistance of GBM [108,109,110,111]. In this regard, the significant decrease in p73 might be crucial for the increase in polyploidization of tumor cells as was recently suggested [112,113] and confirmed by us in radioresistant NSCLC lines [114]. Though we did not investigate polyploidy in the present study, we can forecast the significant reduction of the ploidy abnormality in the LN-229 cell line because of significant upregulation of p73 expression (Figure 8f) in these cells. Conversely, significantly lower basal and IR-induced p73 expression should elicit higher polyploidization in U-87 cells. The irradiation/drug-induced polyploidy facilitates a reversal of senescence and increased survival of the polyploid cells before they go back to diploidy and mitosis [115,116,117]. Albeit polyploidization and multinucleation are different processes, their possible concerted action along with SIPS traits highly likely ensures fitness and illicit survival of cell reproductive capacity, ending in the development of the relapse population as an effect of the adaptation response of the U-87 cell line to IR stress. 

## 4. Materials and Methods

### 4.1. Cell Culture

U-251, LN-229, and U-87 cell lines obtained from ATCC were cultured in DMEM (Gibco, Thermo Fisher Scientific, Waltham, MA, USA) containing 10% FBS (BioloT, Saint Petersburg, Russia) 1% L-glutamine (Gibco, Grand Island, NY USA) and 1% penicillin/streptomycin (Sigma-Aldrich, St. Louis, MO, USA). Cells were maintained in a 5% CO2 humidified incubator at 37 °C.

### 4.2. Irradiation

U-251, LN-229, and U-87 cells were exposed to acute single doses of 2 Gy, 4 Gy, and 6 Gy of X-ray irradiation at room temperature using a 200 kV X-ray RUB RUST-M1 X-irradiator (Ruselectronics, JSC, Moscow, Russia). Non-irradiated cells were used as control.

### 4.3. Anchorage-Dependent Growth Assay

Immediately after irradiation, cells were seeded in 60 mm Petri dishes (at a concentration of 4 × 10^2^ cells/20 cm^2^) for each single dose. Then, cells were incubated at 37 °C for two weeks, fixed with 95% methanol for 15 min, and stained with 10% Giemsa (PanEco, Moscow, Russia) for 15 min at room temperature. Colonies of ≥ 50 cells were manually counted under a light microscope.

Plating efficiency (PE) and survival fraction (SF) values were calculated using the following equations:

PE = (#of colonies observed)/(#of cells plated) × 100(1)

SF = (#of colonies counted)/(#of cells plated × PE) × 100(2)

### 4.4. Soft Agar Clonogenic Assay (Anchorage-Independent Soft Agar)

Six-well plates were coated with 0.6% noble agar (Sigma-Aldrich, St. Louis, MO, USA) pre-warmed to 43 °C in complete medium (1.5 mL agar/9.6 cm^2^) and allowed to solidify at room temperature for 30 min. Immediately after irradiation, cells were collected and cell concentrations were adjusted to 1 × 10^3^ cells/mL. Cell/0.3% noble agar mixtures were added into each well and allowed to solidify for another 30 min at room temperature before placing into a 37 °C humidified cell culture incubator. One hundred µL of complete medium was added on top twice weekly to prevent desiccation of agar. Three weeks later, colonies were stained with 1 mL of 5% crystal violet solution and their number was counted manually.

### 4.5. Wright–Giemsa Analysis of MGCCs 

Cells were seeded in a 96-well plate (at a concentration of 100 × 10^3^ cells/0.32 cm^2^_)_. Twenty-four hours after irradiation, cells were fixed in absolute methanol for 5 min and stained with diluted Wright–Giemsa solution in 1XPBS (pH 6.6) at 1:10 for 1 h, followed by a 1XPBS (pH 6.6) rinse and a distilled water rinse. After that, cells were air-dried and MGCCs were counted manually under a light EVOS™ microscope (Thermo Fisher, Carlsbad, CA, USA).

### 4.6. MTT Assay

For assessing the cell viability, cells were seeded in a 96-well plate (at concentrations of 2 × 10^3^/0.32 cm^2^ and 4 × 10^3^/0.32 cm^2^). Then, 24 h, 48 h, and 72 h after irradiation, 3-(4,5-Dimethylthiazol-2-yl)-2,5-diphenyltetrazolium bromide (MTT) labeling reagent (Sigma-Aldrich, St Louis, MO, USA) (final concentration 0.5 mg/mL) was added into each well. Two hours after incubation in a humidified atmosphere of 5% CO_2_ at 37 ◦C, MTT solution was removed and 150 µL of DMSO was added into each well to extract the blue MTT–formazan crystals. The absorbance measurement was performed at a 570 nm wavelength using a CLARIOstar reader (BMG LABTECH, Ortenberg, Germany). The collected data were analyzed using MARS software (BMG LABTECH, Ortenberg, Germany). 

### 4.7. AlamarBlue Cell Viability

First, 0.6% agar/medium mixtures were added to each well of a 96-well plate (100 µL agar/0.32 cm^2^) and allowed to solidify for 30 min at room temperature. Immediately after irradiation, cells were collected, and cell suspensions were gently mixed with 0.2% noble agar pre-warmed to 43 °C. Cells (at concentrations of 6 × 10^2^ cells/100 μL, 12 × 10^2^ cells/100 μL, 24 × 10^2^ cells/100 μL, and 48 × 10^2^ cells/100 μL) were seeded into each well. After solidification, 50 µL of 0.3% noble agar in complete medium was added into each well as a feeder layer and allowed to solidify for 30 min at room temperature. Cells were maintained in a 5% CO2 humidified atmosphere at 37 °C for one week. After that, 10% (*v*/*v*) alamarBlue ® cell viability reagent (Invitrogen, Carlsbad, CA, USA) was added into each well and plates were maintained for 2 h in a 5% CO_2_ incubator at 37 °C. Absorbance was measured using a CLARIOstar (BMG LABTECH, Ortenberg, Germany) at an excitation/emission wavelength of 530/590 nm. Analysis of data was performed using MARS software (BMG LABTECH, Ortenberg, Germany).

### 4.8. Migration Assay (Scratch Wound Test)

U-251, LN-229, and U-87 cells were seeded in a 96-well plate (at a concentration of 25 × 10^4^ cells/0.32 cm^2^) and maintained in a 5% CO2 humidified atmosphere at 37 °C to form a monolayer with confluence. Immediately after irradiation, the cell monolayer was scratched in a straight line with a 200 μL sterile micropipette tip and washed with 1XPBS (pH = 7.4) 3 times to remove cell debris. Cells were incubated with complete culture medium for another 96 h. The initial areas of the wounds were imaged at time zero (t = 0 h) and after scratching at 24 h, 48 h, 72 h, and 96 h (t = ∆ h) using the ImageXpress Micro XL System (Molecular Devices LLC, San Jose, CA, USA). The percentage of migratory ability was assessed using the following equation:

% of wound healing = [(at = 0 h – at = ∆ h)/at = 0 h],(3)

where at = 0 h is the area of wound calculated immediately after wounding, and at = Δ h is the wound healing area calculated at 24 h, 48 h, 72 h, and 96 h after wounding.

### 4.9. Click-iT™ EdU Alexa Fluor 488 (Cell Proliferation Assay)

Cells (at concentrations of 15 × 10^2^ cells/0.32 cm^2^ and 2 × 10^3^ cells/0.32 cm^2^) were seeded in a 96-well plate for 72 h. Then, 5-ethynyl-2-deoxyuridine (EdU) labeling reagent (final concentration 10 μM) was added to cell cultures and maintained in a 5% CO_2_ humidified incubator at 37 °C for 2.5 h. Then, cells were fixed in 2% (*v*/*v*) paraformaldehyde at room temperature for 15 min and incubated with 6 μg/mL Hoechst 33,342 (Thermo Fisher Scientific, Waltham, MA, USA) overnight for nuclear staining. Following two rinses in 1XPBS, 1XEdU buffer additive was added for 1 h and incubated at room temperature protected from light. Imaging and analysis of proliferating cells were performed using the ImageXpress Micro XL High-Content Screening System (Molecular Devices LLC, San Jose, CA, USA). 

### 4.10. Immunofluorescence Analysis of Ki-67 

Cells (at a concentration of 2 × 10^3^ cells/0.05 cm^2^) were seeded in a 384-well plate for 24 h. Then, cells were washed briefly in 1XPBS (pH 7.4) and fixed with 4% formaldehyde for 15 min, followed by 3 rinses in 1X PBS (pH 7.4). After that, cells were permeabilized with 0.25% Triton X-100 for 15 min and then washed 3 times in 1X PBS (pH 7.4). After blocking cells with 6% bovine serum albumin (BSA) (Sigma-Aldrich, St. Louis, MO, USA) in 1X PBS (pH 7.4) for 1 h at room temperature, cells were incubated with mouse monoclonal Ki-67 antibody (5 μg/mL, clone Ki-S5, Sigma-Aldrich, Darmstadt, Germany) diluted in 1X PBS with 1% BSA and 0.3% Triton X-100 for 1 h at room temperature. After 3 rinses in PBS, cells were incubated for 1 h at room temperature with Alexa Fluor 555 goat anti-mouse secondary antibody (1:500, Merck-Millipore, Burlington, VT, USA) diluted in 1X PBS with 1% BSA and 0.3% Triton X-100. Nuclei were counterstained with Hoechst 33342 solution (dilution 6 μg/mL, Thermo Scientific, Rockford, IL, USA). Cells were imaged and inner integrated fluorescence intensities of cells were calculated using the ImageXpress XL fluorescence microscope (Molecular Devices LLC, San Jose, CA, USA). 

### 4.11. Apoptosis Assay 

To quantify the proportion of early-stage apoptotic cells, the commercial kit “Membrane Permeability/Dead Cell Apoptosis Kit with YO-PRO^®^-1 and PI for Flow Cytometry” (Molecular Probes, Eugene, OR, USA, Catalog Number: V13243) was used. The cells were stained according to the manufacturer’s protocol. Twenty-four hours after radiation, cells were collected and washed in cold phosphate-buffered saline (PBS). One microliter of YO-PRO^®^-1 stock solution and 1 μL of PI stock solution were added to each 1 mL of cell suspension. Cells were incubated on ice for 20–30 min. Cells were analyzed by flow cytometry (BD FACSCalibur, BD Biosciences, San Jose, CA, USA) using 488 nm excitation with green fluorescence emission for YOPRO^®^-1 (i.e., 530/30 bandpass) and red fluorescence emission for propidium iodide (i.e., 610/20 bandpass).

### 4.12. Analysis of Senescence-Associated β-Galactosidase-Positive Cells

The fraction of senescence-associated β-galactosidase (SA-beta-Gal)-positive cells was analyzed using the “Cellular Senescence Assay” commercial kit (EMD Millipore, Burlington, MA, USA, Catalog Number: KAA002). The cells were stained according to the manufacturer’s protocol. The stained cells were visualized using an EVOS^®^ FL Auto Imaging System (Fisher Scientific, Pittsburgh, PA, USA) with 20× objective. The proportion of SA-β-Gal-positive cells was calculated manually.

### 4.13. Western Blotting

Twenty-four hours after irradiation, cell lysates were prepared using radioimmunoprecipitation assay (RIPA) buffer. The protein concentrations were determined using BCA assay (Thermo Scientific™ Pierce™ BCA Protein Assay Kit, Rockford, IL, USA). Thirty μg of lysates was mixed with 4X SDS loading buffer, heat denatured at 95 °C for 5 min, and separated by 8–16% SDS-PAGE (Bio-Rad Laboratories, Mini-PROTEAN TGX Gels, Hercules, CA, USA). Proteins were transferred onto nitrocellulose membranes (7.1 × 8.5 cm, Bio-Rad Laboratories, Neuberg, Germany). Following blocking (Pierce™ Protein-Free Blocking Buffer, Thermo Scientific™ Waltham, MA, USA), the membrane was incubated with primary anti-vimentin antibody (1 µg/mL, ab45939, Abcam, Cambridge, MA, USA), anti-E-cadherin antibody [EP700Y] (1:1000, ab40772, Abcam, Cambridge, MA, USA), anti-N-cadherin antibody (1:2500, aa. 802-819, BD Transduction Laboratories™, San Jose, CA, USA), anti-FOSL-1 antibody (0.5 µg/mL, SAB2108461-100UL, Sigma-Aldrich, Darmstadt, Germany), anti-p63 antibody (1:1000, ab124762, Abcam, Cambridge, MA, USA) and anti-p73 antibody (1:2000, ab40658, Abcam, Cambridge, MA, USA) at 4 ◦C overnight. Following 3 rinses in PBST for 3 min each, the membrane was incubated with anti-rabbit IgG-HRP conjugate (1:5000, IMTEC, Moscow, Russia) and anti-mouse IgG-HRP conjugate (1:1000, IMTEC Ltd., Moscow, Russia) antibodies for 1 h at room temperature. Blotting bands were detected by Clarity™ Western ECL Substrate reagent (Bio-Rad, Hercules, CA, USA). The resulting data were normalized by staining membranes with Ponceau S solution. The relative densities of bands were calculated using Image Lab Software 4.1.

### 4.14. Statistical Analysis

Statistics were performed using the GraphPad Prism 9.0.2.161 (GraphPad Software, San Diego, CA, USA) software and Excel 2010 (Microsoft, Redmond, WA, USA) software. Statistical significance was tested using a Mann–Whitney U test. *p*-values ≤ 0.05 were considered statistically significant. Data are presented as the mean ± SEM.

## 5. Conclusions

We demonstrated significant enrichment in MGCCs, slow-cycling quiescent populations of three GBM cell lines, and generation of numerous small mononuclear (RJ) U-87-derived cells that eventually form cell colonies, a process termed neosis, in response to acute X-ray irradiation at therapeutic doses of 2–6 Gy. Our added value on the expression of p73 and p63 in ensuring the SIPS and polyploidy of GBM cells has not yet been fully elucidated. Consequently, more thorough identification and characterization of the p53/p63/p73–*PTEN* network in GBM may render important insights into IR-induced EMT/pEMT/UJT and tumorigenesis. Additionally, we propose that IR-induced attenuation of UJT and subsequent metabolic dormancy seem to be characteristic of the highest clonogenic survival and fitness underlying the highest radioresistance of the *PTEN*-deficient *TP53wt* U-87 cell line. The radioresistance of GBM emphasizes the need for better understanding of the invasion–metastasis mechanisms and especially those that target GBM stress-adaptive polyploidization/multinucleation and stemness. 

## Data Availability

The materials and data are available from the corresponding author.
